# Mortality Rates after Tuberculosis Treatment, Georgia, USA, 2008–2019

**DOI:** 10.3201/eid3011.240329

**Published:** 2024-11

**Authors:** Sarah Gorvetzian, Antonio G. Pacheco, Erin Anderson, Susan M. Ray, Marcos C. Schechter

**Affiliations:** Emory University School of Medicine, Atlanta, Georgia, USA (S. Gorvetzian, S.M. Ray, M.C. Schechter); Fundaçāo Oswaldo Cruz, Rio de Janeiro, Brazil (A.G. Pacheco); Georgia Department of Public Health, Atlanta (E. Anderson, S.M. Ray, M.C. Schechter)

**Keywords:** tuberculosis, mortality, HIV, bacteria, HIV/AIDS and other retroviruses, respiratory infections, tuberculosis and other mycobacteria, Georgia, United States

## Abstract

Limited data exist on mortality rates after tuberculosis (TB) treatment in the United States. We analyzed mortality rates for all adults in Georgia, USA, who had a TB diagnosis and finished treatment during January 1, 2008–December 31, 2019. We obtained posttreatment mortality rate data from the National Death Index and calculated standardized mortality ratios (SMRs) for TB treatment survivors and the general Georgia population. Among 3,182 TB treatment survivors, 233 (7.3%) had died as of December 31, 2019. The overall TB cohort age- and sex-adjusted SMR was 0.89 (95% CI 0.73–1.05). The SMR among US-born TB treatment survivors was 1.56 (95% CI 1.36–1.77). In the TB cohort, US-born status, HIV co-infection, excess alcohol use, diabetes mellitus, and end-stage renal disease were associated with increased risk for death after TB treatment. TB treatment survivors could benefit from improved linkage to primary and HIV comprehensive care to prevent posttreatment death.

Tuberculosis (TB) is a leading cause of death globally (*1*). However, TB-related mortality rate estimates usually do not account for posttreatment deaths (*1*). TB survivors are at increased risk for disability and death after treatment completion (*2*–*6*). The End TB Strategy focuses on TB prevention, diagnosis, and treatment but does not directly address posttreatment mortality rates (*7*), a critical omission because the number of TB survivors alive in a 2020 study was shown to be >10 times the global TB incidence (*8*).

A 2019 meta-analysis found a pooled standardized mortality ratio (SMR) of 2.91 among persons who completed TB treatment compared with control groups (*4*). Only 1 US-based study has reportedly measured posttreatment mortality rates among persons from Texas, Massachusetts, and Washington treated during 1993–2002 (*9*). That study found a higher all-cause death rate among persons who completed treatment for active TB (20.6%) than among those treated for latent TB (3.1%); however, that study did not compare rates with those from the general population, report causes of death, or investigate individual-level risk factors for posttreatment death, aside from demographics and HIV status (*9*).

Understanding long-term outcomes among TB survivors is needed to inform post-TB care policies in Georgia and in the rest of the United States. We performed survival analyses to determine risk factors for posttreatment deaths among persons who survived TB treatment. We also performed SMR analysis to investigate whether TB treatment survivors have a higher risk for death than the general state population in Georgia (i.e., persons who had no TB). We did not adjust for individual-level medical characteristics, such as other illnesses, because those data are not available for the entire Georgia population. Institutional Review Boards of Emory University and the Georgia Department of Public Health (GDPH) in Atlanta, Georgia, USA, approved this study.

## Methods

We included in the study persons who had a TB diagnosis in Georgia, USA, during January 1, 2008 (earliest date TB data were available) to December 31, 2019 (last date long-term mortality data were available). We included patients who were >18 years of age at the time of TB diagnosis and those who ended treatment by December 31, 2019, and had bacteriologic or clinical TB diagnoses along with pulmonary or extrapulmonary disease. Because we were investigating posttreatment deaths, we excluded persons who died before or during TB treatment and those who had missing treatment dates. However, we included persons who did not complete TB treatment but were alive when treatment ended. We also excluded persons with insufficient identifiers to query the Centers for Disease Control and Prevention (CDC) National Death Index (NDI) (*10*). TB is a notifiable disease in Georgia, and directly observed therapy is the standard of care (*11*). Incentives, such as housing and food vouchers, are generally available to persons with TB during but not after treatment. Similar to current US guidelines, no guidance exists for posttreatment care in Georgia (*12*,*13*).

### Data Sources

We obtained all TB data by using the GDPH’s electronic records (hereafter TB database) (*14*). Whenever applicable, we reported which data were missing and for how many subjects. We obtained posttreatment death data from the NDI (*10*). The NDI assigns a probabilistic score of 1–5 by using matching identifying data, and we considered scores of 1–3 as true matches according to NDI guidance (*10*). We reviewed all matches to ensure a plausible timeline existed between treatment completion and death.

We obtained mortality data for residents of Georgia who were >18 years of age and did not have TB during 2008–2019 (hereafter Georgia population) by using GDPH’s Online Analytical Statistical Information System (*15*). We collected prevalence data for diabetes (*16*), alcohol use (*17*), HIV infection (*18*), homelessness (*19*), and non–US-born residents (*20*) in Georgia from public databases; we show data from our study midpoint (2014) or from the closest timepoint, if 2014 data were not available. Tobacco use data were unavailable in the TB database.

### Study Definitions

We classified TB cases as culture confirmed if >1 culture from any site was positive for *Mycobacterium tuberculosis*, as clinical if no positive culture was obtained, or as other if a positive acid-fast bacilli smear or nucleic acid amplification test was obtained without a positive culture. We classified cavitary and miliary disease on the basis of chest radiograph and chest computerized tomography data, when available.

We identified persons with >1 TB episode during the study period by searching the TB database for duplicated dates of birth, names, or Social Security identification numbers. We manually reviewed all potential duplicates; when >1 TB episode was observed, we used characteristics that manifested during the first TB episode as baseline data.

We classified TB treatment outcomes as either complete or incomplete. Reasons for incomplete treatment were being lost to follow-up, having adverse treatment events, declining treatment, or moving out of Georgia during treatment. Treatment failure as defined by the World Health Organization TB treatment outcome classification (i.e., *M. tuberculosis*–positive sputum cultures after >4 months of treatment) is not recorded in the Georgia TB database. We defined the person-time follow-up for the TB cohort as the difference between the treatment stop date (irrespective of treatment completion) and either death or the study end date (December 31, 2019), whichever came first. We defined follow-up time for the Georgia population as the sum of the adult population in Georgia for each study year (2008–2019). We calculated mortality rates for the Georgia population from 2008–2019 by dividing the total number of deaths each year by that year’s total population.

The NDI reports patient-level causes of death and the Online Analytical Statistical Information System reports the proportion of deaths in the Georgia population according to codes from the International Classification of Diseases, 10th Revision (ICD-10). We grouped causes of death according to ICD-10 codes as follows: cardiovascular disease (I09.X–I80.X), HIV (B20.X–B24.X), malignancy (C01.X–C34.X), respiratory disease (A16.X, A31.X, J18.X–J69.X), trauma/poisoning (V.X, W.X, X.X), and all others.

### Data Analysis

We performed analyses in R version 4.1.2 (The R Project for Statistical Computing, https://www.r-project.org). We reported continuous data as medians and interquartile ranges (IQRs) or means and SDs, where appropriate. We restricted the following analyses to the TB cohort and only included persons who survived TB treatment. We calculated posttreatment death rates stratified by demographic, medical, and TB diagnosis and treatment characteristics. Because persons co-infected with TB and HIV and US-born persons had higher death rates, we calculated the rates of HIV co-infection and births in the United States stratified by demographic, medical, and TB diagnosis and treatment characteristics. We then used Kaplan-Meier curves to depict survival after stratifying TB treatment according to HIV status and place of birth; we calculated p values by using the log-rank test. We censored persons at death or on December 31, 2019 (the last date NDI data are available), whichever came first. Finally, we used Cox proportional hazard models to estimate the relationship between individual-level characteristics and posttreatment deaths. We verified that all models met proportional hazards assumptions. We selected the multivariable Cox proportional hazard final model according to the best fit of the Akaike Information Criterion (AIC) (*21*). The AIC is a model selection method that penalizes the usual goodness of fit measurement by 2 times the number of estimated parameters to avoid overfitting; the lower the AIC, the better the model.

To compare the age of death between TB treatment survivors and the overall Georgia population, we calculated the standardized mortality rate per 1,000 person-years. We defined the age of persons with TB as their age at the time of death or as of December 31, 2019, whichever came first. We used the CDC 2000 projected US population as the standard population (*22*), and we calculated standard populations for sex and age by using US census projection reports (*23*). We calculated odds ratios and 95% CIs to compare causes of death between TB treatment survivors and the general population. We followed the Strengthening the Reporting of Observational Studies in Epidemiology guidelines for observational cohort studies (*24*). 

## Results

### Baseline Characteristics of TB Cohort Patients and Georgia Population

**Figure 1:**
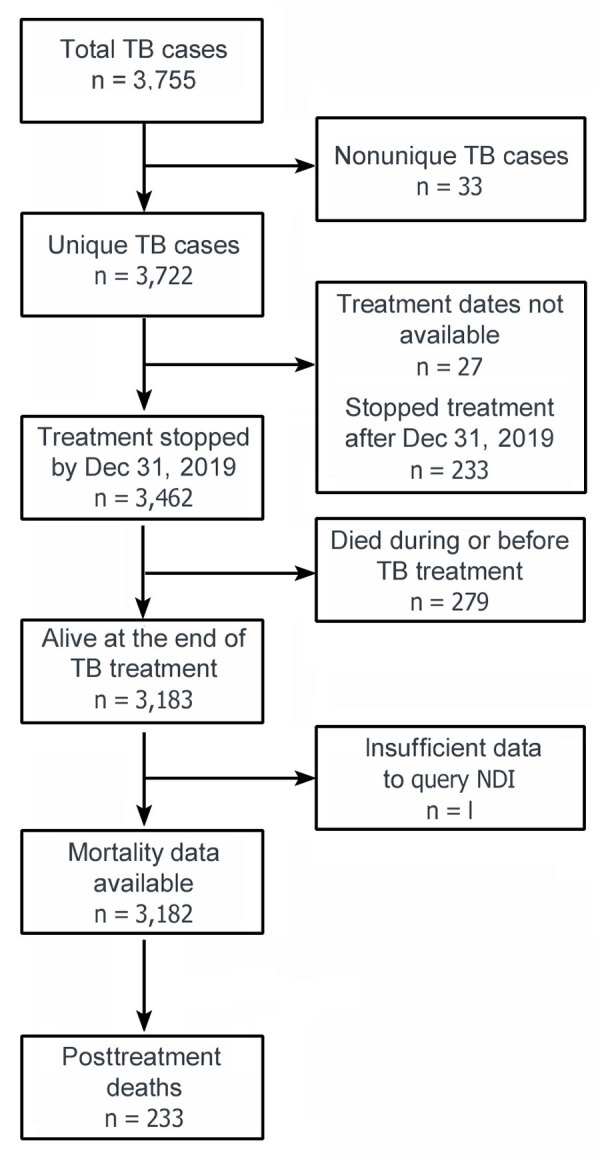
Flow chart of populations in study of mortality rates after TB treatment, Georgia, USA, 2008–2019. Persons who had a TB diagnosis in Georgia during January 1, 2008–December 31, 2019, were included in the study and compared with the general Georgia population. NDI, National Death Index; TB, tuberculosis.

A total of 3,755 TB episodes among 3,722 unique persons were diagnosed in Georgia during the study period (Figure 1). Among the 3,722 patients, reasons for exclusion from the study were as follows: treatment dates were not available (n = 27), treatment ended after December 31, 2019 (n = 233), death occurred before or during treatment (n = 279), and insufficient data was available to query the NDI (n = 1). We included 3,182 patients in the TB cohort. The median age at TB diagnosis was 44 years (IQR 32–57 years); a total of 2,093 (66%) patients were male, 1,089 (34%) female, 1,625 (51%) non–US-born persons, and 420 (13%) non-Hispanic White persons (Table 1). During the same period, the average age of the overall Georgia population was 45 years (IQR 31–58 years); 48% were male, 52% female, and 57% non-Hispanic White persons. In 2016, a total of 10% of Georgia residents were non–US-born persons. The prevalence of several co-existing illnesses or risk factors was higher in the TB cohort than in the overall Georgia population in 2014 (our study midpoint), including HIV (10% vs. 0.5%), homelessness (10% vs. 0.12%), excess alcohol use (15% vs. 5.3%), and diabetes mellitus (12% vs. 11%).

**Table 1:** Characteristics of patients who finished TB treatment stratified according to posttreatment death, HIV infection, and place of birth in study of mortality rates after TB treatment, Georgia, USA, 2008–2019*

Characteristics	No. (%) patients			
	Overall cohort	Posttreatment deaths	HIV positive	US-born persons
Total no. patients	3,218	233	328	1,557
Age, y				
18–44	1,595 (50)	32 (2)	183 (11)	581 (36)
45–64	1,147 (36)	114 (10)	135 (12)	728 (63)
>65	440 (14)	87 (20)	10 (2)	248 (56)
Sex				
F	1,089 (34)	70 (6)	93 (9)	477 (44)
M	2,093 (66)	163 (8)	235 (11)	1,080 (52)
Place of birth				
Non–US-born	1,625 (51)	32 (2)	113 (7)	NA
US-born	1,557 (49)	201 (13)	215 (14)	NA
Ethnicity				
Non-Hispanic White	420 (13)	59 (14)	14 (3)	368 (88)
Non-Hispanic Asian	669 (21)	21 (3)	18 (3)	19 (3)
Non-Hispanic Black	1,479 (46)	142 (10)	247 (17)	1,112 (75)
Hispanic, all races	559 (18)	11 (2)	40 (7)	29 (5)
Other	55 (2)	0 (0)	9 (16)	29 (53)
HIV status				
Negative	2,729 (86)	180 (7)	NA	1,292 (47)
Positive	328 (10)	38 (12)	NA	215 (66)
Missing data	125 (4)	15 (12)	NA	50 (40)
Injection drug use				
No	3,097 (97)	227 (7)	306 (10)	1,509 (49)
Yes	36 (1)	4 (11)	14 (39)	33 (92)
Missing data	49 (2)	2 (4)	8 (16)	15 (31)
Excess alcohol use				
No	2,659 (84)	162 (6)	257 (10)	1,169 (44)
Yes	467 (15)	69 (15)	65 (14)	370 (79)
Missing data	56 (2)	2 (4)	6 (11)	18 (32)
Homeless within year before TB diagnosis				
No	2,860 (90)	187 (7)	246 (9)	1,294 (45)
Yes	313 (10)	45 (14)	81 (26)	262 (84)
Missing data	9 (<1)	1 (11)	1 (11)	1 (11)
Diabetes mellitus				
No	2,791 (88)	178 (6)	319 (11)	1,368 (48)
Yes	391 (12)	55 (14)	9 (2)	189 (49)
End-stage renal disease				
No	3,139 (99)	224 (7)	324 (10)	1,527 (49)
Yes	43 (1)	9 (21)	4 (9)	30 (70)

*NA, not applicable; TB, tuberculosis.

### TB Manifestations and Treatment Characteristics

Most (n = 2,391 [75%]) TB cases were culture confirmed. Pulmonary disease occurred in 2,551 (80%) cases and extrapulmonary disease in 631 (20%) cases; both pulmonary and extrapulmonary disease occurred in 253 (8%) cases. Among patients who had available drug susceptibility results, 2,077 (88%) of those had rifampin- and isoniazid-susceptible TB. The median TB treatment duration was 224 days (IQR 189–289 days); 211 (7%) patients did not complete TB treatment. Reasons for treatment noncompletion included adverse events (n = 11), declined treatment (n = 12), loss to follow-up or moved during therapy (n = 93), and missing data/reason not recorded (n = 93).

### TB Cohort Characteristics Stratified According to Posttreatment Death

Overall, 233 (7%) patients died after completing TB treatment (Table 1). Among 1,557 US-born persons, 201 (13%) died posttreatment compared with 32/1,625 (2%) of non–US-born persons. A total of 59/420 (14%) non-Hispanic White, 142/1,479 (10%) non-Hispanic Black, 21/669 (3%) non-Hispanic Asian, and 11/559 (2%) Hispanic patients died after completing TB treatment. Among 328 persons with TB co-infected with HIV, 38 (12%) died after TB treatment compared with 180/2,729 (7%) of persons who were not co-infected with HIV. Excess alcohol use, homelessness, diabetes mellitus, and end-stage renal disease were associated with a higher risk for posttreatment deaths (Table 1) Posttreatment death rates were the same among patients who completed treatment (218/2,973 [7%]) and those who did not complete treatment (15/211 [7%]) (Table 2).

**Table 2:** TB diagnosis and treatment characteristics stratified according to posttreatment death, HIV infection, and place of birth in study of mortality rates after TB treatment, Georgia, USA, 2008–2019*

Characteristics	No. (%) patients			
	Overall cohort	Posttreatment deaths	HIV positive	US-born persons
Total no. patients	3,218	233	328	1,557
Case verification				
Culture confirmed	2,391 (75)	190 (8)	243 (10)	1,213 (51)
Clinical case	743 (23)	39 (5)	83 (11)	327 (44)
Other†	48 (2)	4 (8)	2 (4)	17 (35)
Site of TB disease				
Pulmonary	2,551 (80)	202 (8)	270 (11)	1,306 (51)
Extrapulmonary TB	631 (20)	31 (5)	58 (9)	251 (40)
Cavitary disease, n = 2,551 patients with pulmonary disease‡				
No	1,513 (59)	116(8)	214 (14)	729 (48)
Yes	1,038 (41)	86 (8)	56 (5)	577 (56)
Sputum smear, n = 2,551 patients with pulmonary disease‡				
Negative	1,254 (49)	95 (8)	137 (11)	650 (52)
Positive	1,192 (47)	98 (8)	124 (10)	600 (50)
Missing data	105 (4)	9 (9)	9 (9)	56 (53)
Drug susceptibility, n = 2,357 patients with available results‡				
RIF/INH susceptible	2,077 (88)	167 (8)	189 (9)	1,029 (50)
RIF susceptible/INH resistant	250 (11)	20 (8)	50 (20)	158 (63)
RIF resistant	30 (1)	1 (3)	3 (10)	10 (33)
Completed tuberculosis treatment				
Yes	2,973 (93)	218 (7)	303 (10)	1,514 (51)
No	211 (7)§	15 (7)	25 (12)	43 (21)

*INH, isoniazid; RIF, rifampin; TB, tuberculosis. †Other comprised positive smear/tissue (n = 6) and positive nucleic acid amplification (n = 42). ‡Number of patients used in denominator to calculate percentage in overall cohort column. §Incomplete treatment included adverse treatment events (n = 11), uncooperative or refused treatments (n = 12), lost to follow-up (n = 87), moved out of jurisdiction (n = 6), or reason was missing (n = 95).

### TB Cohort Characteristics Stratified According to HIV Status and Birth Place

HIV infection occurred among 183/1,595 (11%) patients who were 18–44 years of age, 135/1,147 (12%) patients 45–65 years of age, and 10/440 (2%) patients >65 years of age at the time of TB diagnosis (Table 1). HIV co-infections occurred in 247/1,479 (17%) non-Hispanic Black, 14/420 (3%) non-Hispanic White, 18/669 (3%) non-Hispanic Asian, and 40/559 (7%) Hispanic persons with TB. Among persons who injected drugs, 14/36 (39%) were co-infected with HIV compared with 306/3,097 (10%) patients who did not inject drugs. Among patients who used excess alcohol, 65/467 (14%) had HIV co-infections compared with 257/2,669 (10%) patients who did not use excess alcohol.

A total of 581/1,595 (36%) patients 18–44 years, 728/1,147 (63%) patients 45–64 years, and 248/440 (56%) patients >65 years of age at the time of TB diagnosis were US-born persons. We also found that 368/420 (88%) non-Hispanic White and 1,112/1,479 (75%) non-Hispanic Black patients were US-born persons with TB. Those rates were higher than for non-Hispanic Asian (19/669 [3%]) and Hispanic (29/559 [5%]) patients. US-born persons accounted for most persons who were HIV co-infected (215/328 [66%]), who injected drugs (33/36 [92%]), used excess alcohol (370/467 [79%]), had a history of homelessness (262/313 [84%]), or had end-stage renal disease (30/43 [70%]). Among 211/3,218 (7%) persons who did not complete TB treatment, 43 (21%) of those were US-born (Table 2).

### TB Cohort Survival Analyses

**Figure 2:**
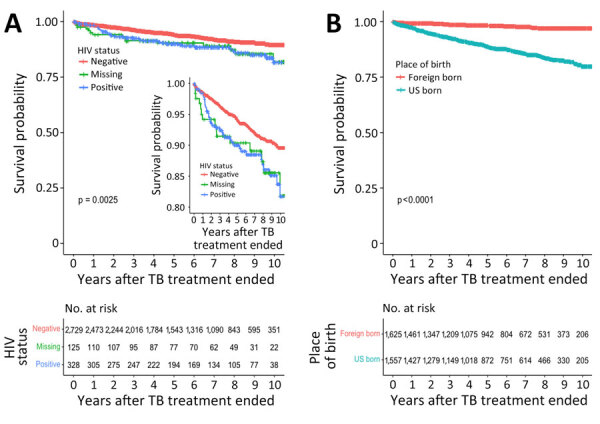
Survival probabilities in study of mortality rates after TB treatment, Georgia, USA, 2008–2019. Kaplan-Meier curves were used to plot survival probabilities of treated persons with TB over a 10-year period after treatment ended, stratified according to HIV status (A) and place of birth (B). Inset in panel A shows detailed curve with probabilities of 0.80–1.00. p values were calculated by log rank test. Number at risk tables below the curves indicate the total number of patients remaining in the study at each time point in each group, including any persons who experienced the event or were censored at that time point. Missing indicates missing data. TB, tuberculosis.

The TB cohort median follow-up time was 5.8 years (IQR 2.8–8.7 years), for a total follow-up time of 18,426 person-years. We used Kaplan-Meier curves to depict posttreatment survival stratified by HIV status and place of birth (Figure 2). In univariate Cox proportional hazard models, older age at TB diagnosis was associated with increased risk for posttreatment death; the hazard ratio (HR) was 1.06 (95% CI 1.05–1.07) per year of age at the time of TB diagnosis (Table 3). Other risk factors for posttreatment death were being US-born (HR 6.68 [95% CI 4.60–9.70]), having HIV-positive (HR 1.70 [95% CI 1.22–2.45]) or missing HIV (HR 1.72 [95% CI 1.02–2.92]) status, using excess alcohol (HR 2.38 [95% CI 1.79–3.15]), having a history of homelessness (HR 2.19 [95% CI 1.58–3.04]), diabetes mellitus (HR 2.83 [95% CI 2.09–3.83]), and having end-stage renal disease (HR 3.36 [95% CI 1.73–6.54]). Extrapulmonary TB was associated with decreased risk for posttreatment death in univariate analysis (HR 0.62 [95% CI 0.43–0.91]). Compared with non-Hispanic White persons with TB, those of all other races/ethnicities had a lower posttreatment death HR. In multivariable Cox proportional hazard models (Table 3), factors associated with posttreatment death were older age at TB diagnosis (HR 1.06 [95% CI 1.05–1.07] per year), being US-born (HR 3.42 [95% CI 2.25–5.19]), HIV-positive status (HR 1.87 [95% CI 1.20–2.90]), excess alcohol use (HR 1.64 [95% CI 1.17–2.30]), missing homelessness history (HR 17.3 [95% CI 2.0–150.0]), diabetes mellitus (HR 2.05 [95% CI 1.44–2.91]), and end-stage renal disease (HR 2.24 [95% CI 1.05–4.80]).

**Table 3:** Hazard ratios for TB cohort groups defined by univariate and multivariable models in study of mortality rates after TB treatment, Georgia, USA, 2008–2019*

Characteristics	Univariate analysis, HR (95% CI)	Multivariate model, HR (95% CI)†
Age, per year	1.06 (1.05–1.07)	1.06 (1.05–1.07)
Sex		
F	Referent	NA
M	1.23 (0.92–1.63)	NA
US-born persons	6.68 (4.60–9.70)	3.42 (2.25–5.19)
Ethnicity‡		
Non-Hispanic White	Referent	NA
Non-Hispanic Asian	0.23 (0.14–0.37)	NA
Non-Hispanic Black	0.67 (0.49–0.91)	NA
Hispanic, all races	0.12 (0.06–0.23)	NA
HIV status		
Negative	Referent	Referent
Positive	1.70 (1.22–2.45)	1.87 (1.20–2.90)
Missing data	1.72 (1.02–2.92)	1.58 (0.90–2.78)
Injection drug use		
No	Referent	NA
Yes	1.44 (0.55–4.00)	NA
Missing data	1.46 (0.36–5.91)	NA
Excess alcohol use		
No	Referent	Referent
Yes	2.38 (1.79–3.15)	1.64 (1.17–2.30)
Missing data	1.30 (0.32–5.26)	1.51 (0.34–6.75)
Homeless within past year		
No	Referent	Referent
Yes	2.19 (1.58–3.04)	1.36 (0.89–2.08)
Missing data	1.81 (0.25–12.90)	17.3 (2.00–150.0)
Diabetes mellitus	2.83 (2.09–3.83)	2.05 (1.44–2.91)
End-stage renal disease	3.36 (1.73–6.54)	2.24 (1.05–4.80)
Case verification		
Culture confirmed	Referent	NA
Clinical case	0.66 (0.47–0.93)	NA
Other	1.12 (0.42–3.01)	NA
Extrapulmonary	0.62 (0.43–0.91)	NA
Cavitary disease, n = 2,511§	1.14 (0.86–1.50)	NA
Sputum smear, n = 2,511§		
Negative	Referent	NA
Positive	1.00 (0.76–1.33)	NA
Missing data	0.82 (0.41–1.63)	NA
Drug susceptibility, n = 2,318		
RIF/INH susceptible	Referent	Referent
RIF susceptible/INH resistant	0.99 (0.62–1.57)	0.89 (0.55–1.49)
RIF resistant	0.49 (0.07–3.47)	0.89 (0.12–6.39)
Incomplete TB treatment	0.83 (0.49–1.40)	NA

*HR, hazard ratio; INH, isoniazid; NA, not applicable; RIF, rifampin; TB, tuberculosis. †Model selected according to best fit of Akaike Information Criterion (*21*). ‡Other category was excluded because no deaths occurred in this group (n = 55). §Cavitary disease and sputum smear were not included in the multivariable models because the overall cohort included patients without pulmonary disease.

### TB Cohort and Georgia Population Mortality Rates

The median age at death for those who died after TB treatment was 64.0 years (IQR 55.7–75.3 years), whereas the average age at death in the Georgia population was 70.2 years. Among the deaths in the TB cohort, most were caused by cardiovascular disease (56/233 [24.0%]) or malignancy (56/233 [24.0%]), followed by HIV infection (22/233 [9.9%]) (Table 4). Pulmonary malignancies (n = 21) accounted for 37.5% of total malignancies. Cardiovascular disease was a more frequent cause of death in the overall Georgia population (30.1%) compared with that in the TB cohort (24.0%). Conversely, HIV infection was a more frequent cause of death in the TB cohort (9.9%) compared with the overall Georgia population (0.05%). The mean age of death among those who died from HIV infection in the TB cohort was 49.6 years. No significant differences were observed for percentages of death from other causes between persons with TB and the general Georgia population.

**Table 4:** Comparisons of causes of death between patients who finished TB treatment and the general population in study of mortality rates after TB treatment, Georgia, USA, 2008–2019*

Categories	No. (%) persons		Odds ratio (95% CI)†	Age at death, y, mean (SD)
	Posttreatment deaths	Georgia population deaths		
Total no. persons	233	900,874	NA	233
Cardiovascular disease	56 (24.0)	271,146 (30.1)	0.73 (0.54–0.99)	67.8 (14.8)
HIV	22 (9.9)	4,789 (0.5)	19.6 (12.6–30.4)	49.6 (11.3)
Malignancy	56 (24.0)‡	193,983 (21.5)	1.15 (0.85–1.55)	64.5 (12.0)
Pulmonary disease	26 (11.2)§	90,880 (10.1)	1.11 (0.74–1.68)	68.7 (14.9)
Trauma/poisoning	16 (6.9)	68,550 (7.6)	0.89 (0.53–1.48)	63.3 (14.3)
Other	57 (24.5)	271,526 (30.1)	0.75 (0.55–1.01)	68.0 (14.2)

*NA, not applicable; TB, tuberculosis. †Odds of dying in posttreatment group compared with the general Georgia population. ‡Pulmonary malignancy, n = 21 (37.5% of total malignancies, 9% of total deaths). §Pulmonary TB/mycobacterial infection, n = 7.

### Standardized Mortality Analyses Comparing Age of Death

The crude mortality rate for the overall TB cohort was 12.69/1,000 person-years (95% CI 11.12–14.3/1,000 person-years) (Table 5) compared with 9.92/1,000 person-years (95% CI 9.90–9.94/1,000 person-years); the Georgia population SMR was 0.89 (95% CI 0.73–1.05). We found similar results in a subgroup analysis restricted to culture-confirmed patients who completed treatment. In a subgroup analysis restricted to US-born persons in the TB cohort, the SMR was 1.58 (95% CI 1.41–1.76) (Table 5).

**Table 5:** Mortality rates and standardized mortality ratios for posttreatment groups compared with the general Georgia population in study of mortality rates after TB treatment, Georgia, USA, 2008–2019*

Groups	Mortality rate (95% CI)	Standardized mortality ratio (95% CI)
Overall TB cohort†		
Crude mortality rate	12.69 (11.12–14.43)	Referent
Mortality rate adjusted for age and sex	9.77 (8.29–11.43)	0.91 (0.75–1.07)
Mortality rate adjusted for age only	10.50 (9.09–12.07)	0.98 (0.84–1.12)
Culture confirmed and treatment completed‡		
Crude mortality rate	14.19 (12.20–16.41)	Referent
Mortality rate adjusted for age and sex	10.92 (9.08–13.03)	1.02 (0.84–1.20)
Non-Hispanic White and Black persons§		
Crude mortality rate	22.84 (19.79–26.23)	Referent
Mortality rate adjusted for age and race	19.73 (15.64–24.56)	1.82 (1.60–2.04)¶
US-born persons only#		
Crude mortality rate	22.51(19.50–25.84)	Referent
Mortality rate adjusted for age and sex	16.93 (14.10–20.15)	1.58 (1.41–1.76)

*All rates are per 1,000 person-years. Georgia population had 900,874 deaths; person-time = 90,796,252 person-years. SMR, standardized mortality ratio; TB, tuberculosis. †Number of patients in TB cohort was 3,182. Overall TB cohort follow-up time was 18,426 person-years (no. deaths = 233). ‡Total number was 2,248. Culture-confirmed TB follow-up time was 12,820 person-years (no. deaths = 182). §Total number was 1,899. Follow-up time was 10,797 person-years (no. deaths = 201). ¶Compared with non-Hispanic White and non-Hispanic Black persons in Georgia. #Total number was 1,557. Follow-up time was 8,929 person-years (no. deaths = 201).

## Discussion

We obtained data for persons who survived TB treatment in Georgia during 2008–2019 to report individual-level risk factors for posttreatment death and compared death rates and causes of death to those in the general Georgia population. This study fills a crucial knowledge gap because mortality rates after TB treatment in the United States were only reported >20 years ago (*9*). Among TB treatment survivors, 233/3,182 (7%) died, and the median time between treatment completion and death was 2.9 years. In a survival analysis restricted to the TB cohort, we found that being a US-born patient, living with HIV, excess alcohol use, diabetes mellitus, and end-stage renal disease were significantly associated with post-TB treatment death. In contrast to studies in other countries (*4*,*6*,*25*–*27*), we found no difference between posttreatment mortality rates and mortality rates in the overall population (age- and sex-adjusted SMR of 0.91 [95% CI 0.75–1.07]). However, in a post-hoc analysis, we found that US-born persons with TB had a higher mortality rate (age- and sex-adjusted SMR of 1.58 [95% CI 1.41–1.76]) than the Georgia population.

A landmark meta-analysis (*4*) of 10 studies investigating posttreatment deaths found an SMR of 2.91 (95% CI 2.21–3.84) among TB survivors, a finding that has been replicated by subsequent studies (*4*,*6*,*23*–*25*). Similar to this study, those studies have used the general population as a control (*4*). In this study, the follow-up time (18,426 person-years) was within the range (8,780–13.5 million person-years) of previous studies (*6*,*26*), and the HIV prevalence (10%) was also within the range (1.2%–16.0%) of previous studies (*9*,*29*,*30*). Thus, methodologic and study population differences are unlikely to explain why similar mortality rates existed among TB survivors in our cohort and the general Georgia population. However, increased life expectancy among immigrants in the United States compared with US-born persons (*31*) could explain our SMR findings; the SMR of 1.58 among US-born persons with TB in our cohort aligns with previously published studies.

Being a US-born TB patient was associated with higher rates of posttreatment death in the SMR and survival analyses. The increased death rate among US-born persons in the TB cohort could be, in part, because they were older, were more often HIV co-infected, and had higher rates of excess alcohol use, injection drug use, homelessness, and end-stage renal disease than did non–US-born persons. This finding is consistent with the higher life expectancy among non–US-born persons compared with US-born persons in the United States (*29*). However, it is possible that non–US-born TB treatment survivors emigrated from the United States more frequently than US-born TB treatment survivors, which might affect the mortality rate measurements because the NDI only captures deaths within the United States. We are unaware of US-based emigration data for TB treatment survivors, and challenges exist linking those data to minoritized populations within the NDI database because of higher rates of missing Social Security numbers (*32*) and differences in access to end-of-life care among immigrants (*33*). Those 2 limitations could lead to an undercount of posttreatment deaths among non–US-born persons. However, the other US-based study on mortality rates after TB treatment had a similar percentage of non–US-born persons compared with persons with TB identified in this study (58% vs. 51%) and also found a higher cumulative death rate among US-born persons (37%) compared with non–US-born persons (9%) (*9*). Other studies have also shown that co-existing illnesses are associated with increased posttreatment death rates (*6*,*26*,*34*). For example, a California-based study found that diabetes, HIV co-infection, and end-stage renal disease were associated with an increased death HR 1 year after TB diagnosis compared with age- and sex-matched control patients without TB (*34*). However, that study did not specifically measure TB mortality rates after treatment completion and did not stratify deaths by place of birth. 

Persons with TB/HIV co-infection had a posttreatment death HR of 1.87 compared with non–co-infected persons (referent 1.0) in the TB cohort. Moreover, HIV-related deaths were ≈20-fold higher among those who died after TB treatment than those in the Georgia population (9.9% vs. 0.5%); TB/HIV co-infected persons also died at a younger mean age than those in the Georgia population (49.6 vs. 70.2 years). Persons with HIV/TB co-infection in the TB cohort were younger than non–co-infected persons but had higher rates of homelessness, excess alcohol use, and injection drug use. We have found that persons co-infected with HIV and TB in Atlanta, Georgia, had low rates of virus suppression after TB treatment (45% had virus suppression 1 year after TB treatment) (*35*), which might explain the poor post–TB treatment outcomes. Our findings suggest that strengthening the HIV care continuum might avert deaths among HIV co-infected TB survivors and should be a care priority after TB treatment.

The first limitation of our study is that we did not have individual-level data for the overall Georgia population. Second, the TB database has limitations, such as insufficient data needed to use the World Health Organization TB outcomes classification, lack of tobacco use data, and using nonstandardized definitions for alcohol use. Third, the NDI does not capture deaths that occurred outside of the United States. The strengths of our study are that TB disease notification is mandatory in Georgia and, thus, it is unlikely we missed TB cases, and we ascertained mortality rates through the NDI, which is the most complete database of deaths in the United States. Our findings might not be generalizable to the entire United States, and further studies that include nationwide data are needed.

In conclusion, we found that US-born TB survivors have higher mortality rates than persons in the general population in Georgia, and HIV, excess alcohol use, diabetes mellitus, and end-stage renal disease are risk factors for death after TB treatment. Currently, no guidance exists for post–TB treatment care in Georgia, and such care was not addressed in the latest American Thoracic Society/CDC/Infectious Diseases Society of America guidelines (*12*,*13*). The 2022 Canada TB standards recommend linkage of posttreatment care to primary care providers for TB survivors (*36*). Our findings support this recommendation because the conditions associated with increased posttreatment death in this study are usually treated by primary and HIV care providers in the United States. Most persons with TB in the United States receive >6 months of treatment, usually under directly observed therapy (*37*). Our findings indicate that, to prevent death after TB, comprehensive care during and after treatment should also consider social determinants of health and co-existing illnesses, which might be more prevalent among US-born persons.
